# Unusual presentation of chronic granulomatous infection of the vulva

**DOI:** 10.11604/pamj.2025.52.47.48652

**Published:** 2025-09-29

**Authors:** Amit Toshniwal, Nayan Mundhada

**Affiliations:** 1 Department of Respiratory Medicine, Datta Meghe Institute of Higher Education and Research, Wardha, Maharashtra, India,; 2 Department of General Surgery, Datta Meghe Institute of Higher Education and Research, Wardha, Maharashtra, India

**Keywords:** Genitourinary TB, donovanosis, infectious disease

## Image in medicine

A 36-year-old female hailing from rural central India presented with a 2-month history of progressive, enlarging, painful, hard swelling over the vulvar region. The patient reported intermittent fever and micturition. She had no history of trauma or constitutional symptoms and denied any history of genital ulcers, recent sexual contact with new partners, or history of sexually transmitted disease. On clinical examination, the mass was firm and non-tender with yellowish discharge over the right labia majora. Surgical excision was performed, and the lesion measured approximately 7x6 cm, with an area of necrosis and sloughing. Histopathological examination confirmed a granulomatous infection with caseating necrosis and no evidence of malignant cells. The patient was started on antitubercular therapy, the postoperative recovery was eventful, and the patient remained asymptomatic. This case highlights the importance of considering unusual presentation of genital tuberculosis, especially in the endemic regions.

**Figure 1:**
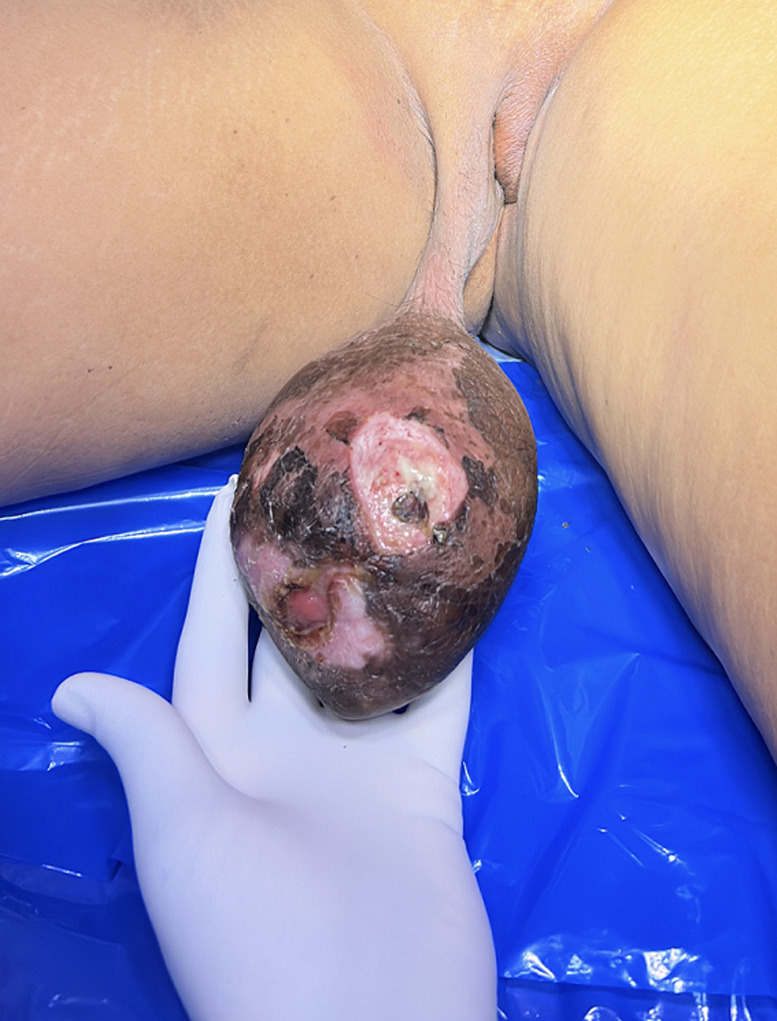
firm non-tender mass over right labia majora with yellowish discharge

